# Compound Fractures

**Published:** 1919-06-28

**Authors:** 


### WAR AND SURGICAL PROGRESS.

### III.—Compound Fractures.

At the International Congress of Surgeons held in Brussels in 1911, where leading men from all over the world were gathered, it was noticeable to an onlooker that opinions on fracture work were grouped under three conflicting headings. There were many who upheld the value of open treatment as showing great advance over any other method. A large section supported the practically exclusive use of apparatus and sought to demonstrate the work of their purely mechanical technique. And, thirdly, a group attempted to point out the failures of both the foregoing methods, to demonstrate the unfortunate occurrences which might follow either of them, maintaining, however, that in the greater number of cases the best results were to be obtained by ensuring immobility, correct position through the use of sandbags, etc., and thus minimising circulatory disturbance due to pressure. By these last early massage and passive movement were also advocated. Each group readily admitted exceptions to their ruling, but stoutly maintained that their particular outlined routine should be recognised as worthy of general application.

### After the Years of War.

Such different methods of dealing with simple bone lesions have had a marked influence on the technique applied to compound fractures, but, nowadays, although still widely divergent opinions are in fashion, there are certain essential considerations on which relatively close agreement exists.

The appalling death-rate and complications in 1914 and 1915 due to many types of compound fracture are too well known to need description, and the marked improvement which has fortunately occurred is the outcome of gradual evolution. To quote from the American *Medical Bulletin*, where complete reviews of the allied medical progress are made:

> Immediate immobilisation, the use of a Thomas splint or some modification of it, careful splinting so as to allow of no grating of bone ends, the removal of foreign bodies introduced with the projectile, the types of cases which may be safely evacuated and those which should be left behind, and the importance of not disturbing injured parts any more than is imperative are all subjects upon which there is substantial agreement. Other questions, such as the character and extent of operative interference, whether antiseptics should be used at all, and, if employed, which one is best, and the kind of splint which should be used after the case leaves the casualty clearing station are yet matters of varying opinion.

In the later days of the War the utilisation of specialised hospitals increased, and although each one had its trained staff and hence to some extent its own particular technique, most of these have been already described by their originators and supporters, and one can here best select a few typical instances of successful progress.

### The Eighth General Hospital.

In the fracture wards of this hospital under the care of Major Sinclair some remarkably good individual and collective results have been obtained. According to his practice immobilisation and drainage have been the main elements in success. *Immediate* immobilisation, a sterile dressing and free drainage, the wound itself being treated *after* a fixed position is secured. In this way the ends of the bones are prevented from opening up fresh paths of infection, and as it is this infection—the organisms—which is the essential danger, he advises that even metallic fragments should be left until they obviously suggest trouble, unless their removal can be effected without any gross disturbance of the tissues. This is all very much opposed to the antiseptic methods such as the Carrel-Dakin which we last described, but there are points of overlapping. Major Sinclair, although opposed to the use of wound antiseptics, produces free drainage and encouragement of fresh serum exudation, etc., by a series of clean cut incisions and multiple rubber tubes kept scrupulously clean and clear. Also he emphasises the importance of noting the character of the discharge as affording valuable clinical data and indications for closure or debridément. Another point he makes is that one should not be too ready to remove detached bony fragments, holding with Sir W. Macewen that bone can grow from bone and not from periosteum alone.

Of course, such procedure will find many critics, but we give it as an example and a highly successful trial of methods running parallel to the early closure of wounds and the relegation to a secondary position of antiseptic treatment. As in all such instances, we feel that the good results obtained depend very largely on the fact that these cases were under observation and control of one set of trained men, one "team," and they masters in their own particular technique. The lead Major Sinclair would apparently give us suggests that with compound war fractures, many of which under the improved conditions of the later years were relatively clean, we should reduce active surgical measures and wound cleansing to a minimum in the first stages at least, but whether such methods can be successfully applied to the corresponding lesions of civil life, street accidents, railway smashes, etc., with people in a less robust condition than the average healthy soldier is a matter to be viewed with expectant interest.

### Extension and Splints.

In previous articles we have described several wound treatments which have practically all been successfully applied to fracture conditions What was then said of the simple wound largely applies to those complicated by bone lesions. There arises, however, the great question of extension and splinting. An apparatus which has received considerable

☞ Previous articles appeared on May 24 and June 7, page 241.

**War and Surgical Progress**—(*continued*).

appreciation is that of Willems. Essentially it consists, as applied to leg bones, of two screws for lateral insertion above the malleoli or knee joint, a bolt on each, with a short attached chain passing downwards to a metal stirrup to which traction is applied. The bolts are approached close to the skin so as to produce a juxta-cutaneous pull, and of the screws themselves it is found they are readily tolerated provided they are firmly fixed in the bone, above the epiphyses. No fracture has ever been recorded during their insertion. Screw extension can be readily combined with suspension as by means of a Thomas splint, and generally the advantages claimed are: 1. The great accuracy and efficiency of traction. 2. The disadvantage and discomfort of pull through one or more articulations is avoided. 3. The surface is left free, facilitating the care of wounds. 4. Mobilisation of all joints is possible throughout the treatment. 5. Over-riding of fragments is prevented and corrections of angular deviations are possible by the use of lateral traction.

The principle employed is by no means new, for direct insertion of pegs, etc., into bone, to facilitate tension, has been frequently described in pre-war days, but we believe that the wide use by the French of this method is the first extensive trial it has received. Our allies have also invented a number of metal extension splints, highly mechanical and spring tension arrangements, for which, although often cumbersome in appearance, great efficiency is claimed. The lessons to be derived, however, with regard to fracture treatment are best taken from the most general aspect of the situation. All the mechanical appliances have largely a common aim, and together with allowing accessibility of wound surfaces, their success or failure largely depends upon their compliance with general requirements.

### Essential Requirements.

A review of many papers from the war areas suggests that, first, simple but efficient extension should be *at once* applied in these cases, in fact at the time the very first surgical dressing is applied. It is not so much a question of the best apparatus, but rather the absence of delay in somehow producing immediate extension. Secondly, if coming under control within the first few hours of injury the whole technique and principle of the primary or delayed suture of wounds should be employed. Fracture departments and wards should be specially equipped and complete in themselves, and it is an invaluable advantage if the patient can be under the personal care of the same set of surgeons throughout. There is perhaps no greater innovation in the present treatment of fractures than in that of simple wounds: the principle is the same throughout. War experience has apparently been of the greatest value in emphasising the absolute necessity that the old maxims of fracture work must be strictly and promptly followed, but combined with thorough appreciation of our means of dealing with actual or potential septic infection.

